# Supplementary material for: Mitochondrial targeted meganuclease as a platform to eliminate mutant mtDNA in vivo
Source: Nat Commun. 2021 May 28;12:3210. doi: 10.1038/s41467-021-23561-7 (PMC8163834; doi:10.1038/s41467-021-23561-7)
Supplement: Supplementary file 1 — Supplemental Information [file 41467_2021_23561_MOESM1_ESM.pdf]

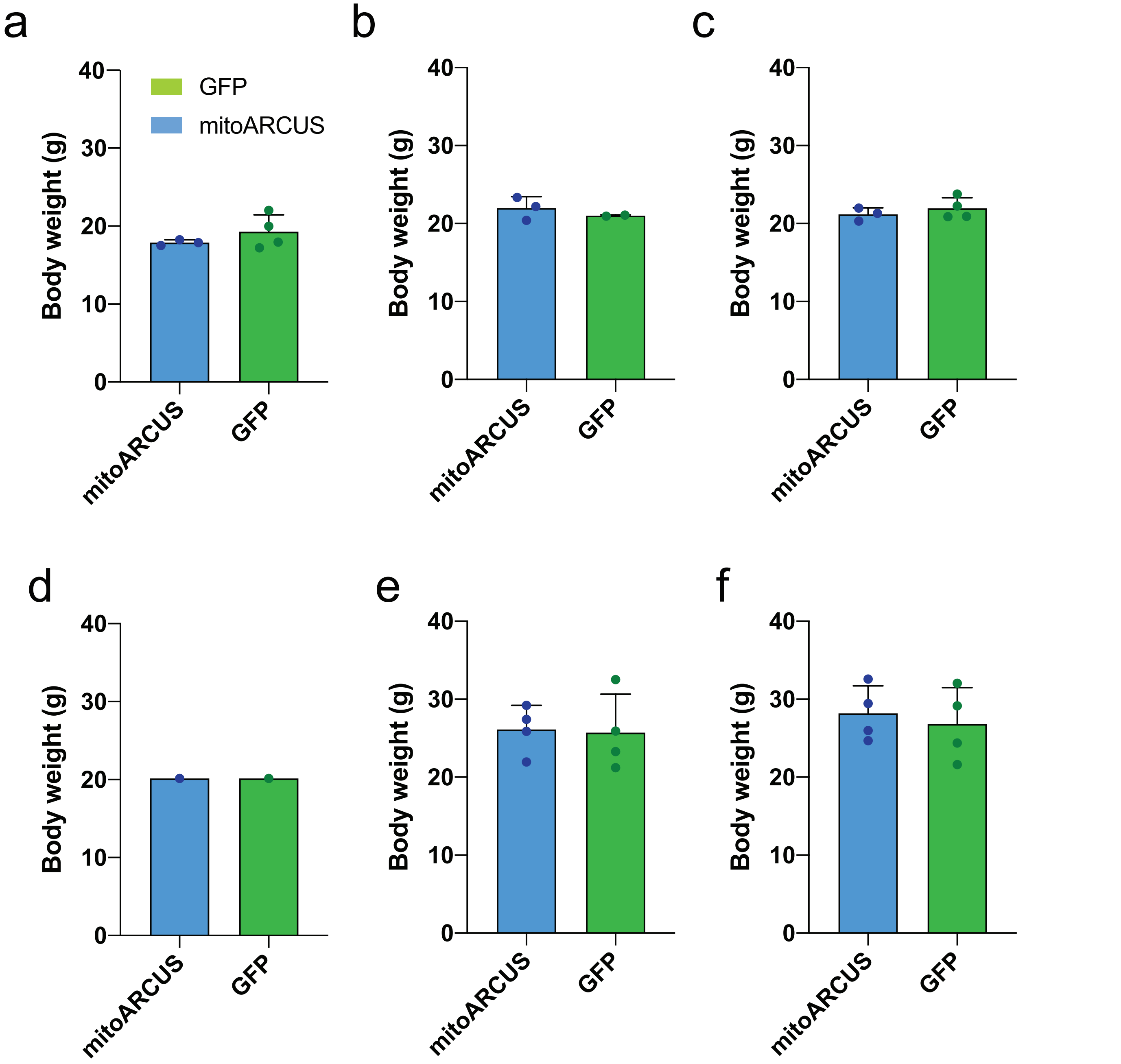

Supplementary Fig S1: Mouse weights of injected animals at 6, 12, and 24 weeks PI. Body weights (grams) of mice injected at 2.5 weeks and sacrificed at (a) 6 weeks PI (n=3 mitoARCUS, n=4 GFP), (b) 12 weeks PI (n=3 mitoARCUS, n=2 GFP), and (c) 24 weeks PI(n=3 mitoARCUS, n=4 GFP). Body weights of mice injected at 6 weeks and sacrificed at (d) 6 weeks PI (n=1), (e) 12 weeks PI (n=4), and (f) 24 weeks PI (n=4). Data are mean ± SEM. Statistical analysis was performed using two-tailed student's t-test when n≥3.

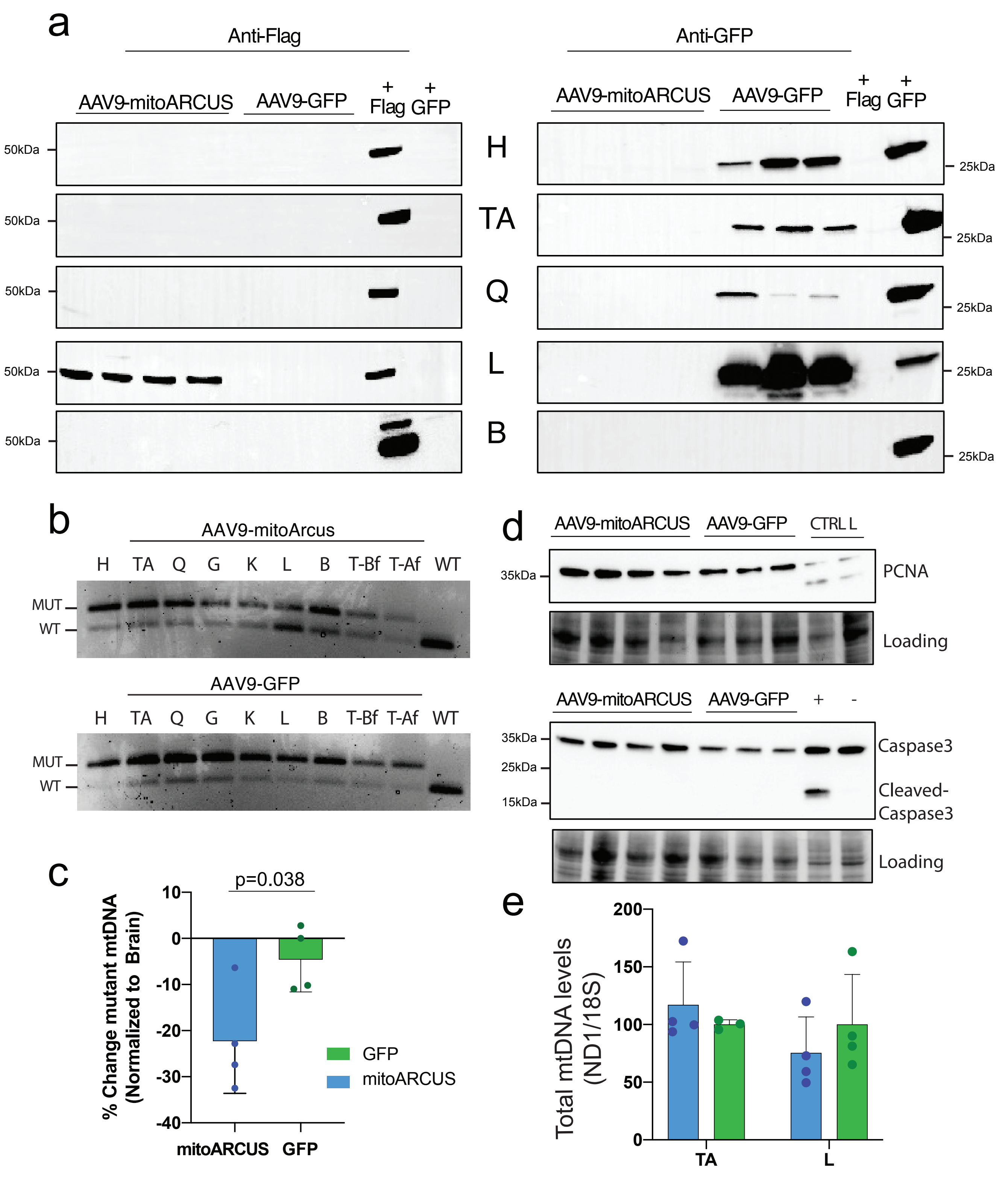

Supplementary Fig S2: **AAV9-mitoARCUS effect in young treated mice 5 days Post Injection** a) Western blots of homogenates probed with Flag antibody for AAV9-mitoARCUS and GFP for AAV9-GFP samples for heart (H), tibialis anterior (TA), quadriceps (Q), liver (L) and brain (B). This experiment was repeated twice with similar results. b) Representative RFLP analysis of DNA samples from an animal injected with AAV9-mitoARCUS and an animal injected with AAV9-GFP 5 days PI in heart (H), tibialis anterior (TA), quadriceps (Q), gastrocnemius (G), kidney (K), liver (L), brain (B), tail before injection (T-Bf), and tail after injection (T-Af). This analyses was performed for each animal. c) Quantification of heteroplasmy shift shown as percent change in heteroplasmy in liver, normalized to brain tissue, from results obtained with RFLP "last cycle hot" PCR analysis (n=4). d) Western blots of liver homogenates of animals injected with AAV9-mitoARCUS (n=4) and AAV9-GFP (n=3). Expression of PCNA shown in blot with control livers from healthy adult mice. Expression of Caspase3 and Cleaved-Caspase3 shown in blot with positive control representing MEFs treated with staurosporine, negative control representing MEFs not treated with staurosporine. Blots are shown along with total protein loading. This experiment was repeated twice. e) Quantification by qPCR of total mtDNA levels were measured in liver (L) and tibialis anterior (TA) at 5 days PI using ND1 mitochondrial primer/probes normalized to 18S (nuclear DNA). n=4, with exception of AAV9-GFP-injected TA (n=3). Data are mean ± SEM. Statistical analysis was performed using two-tailed student's t-test.

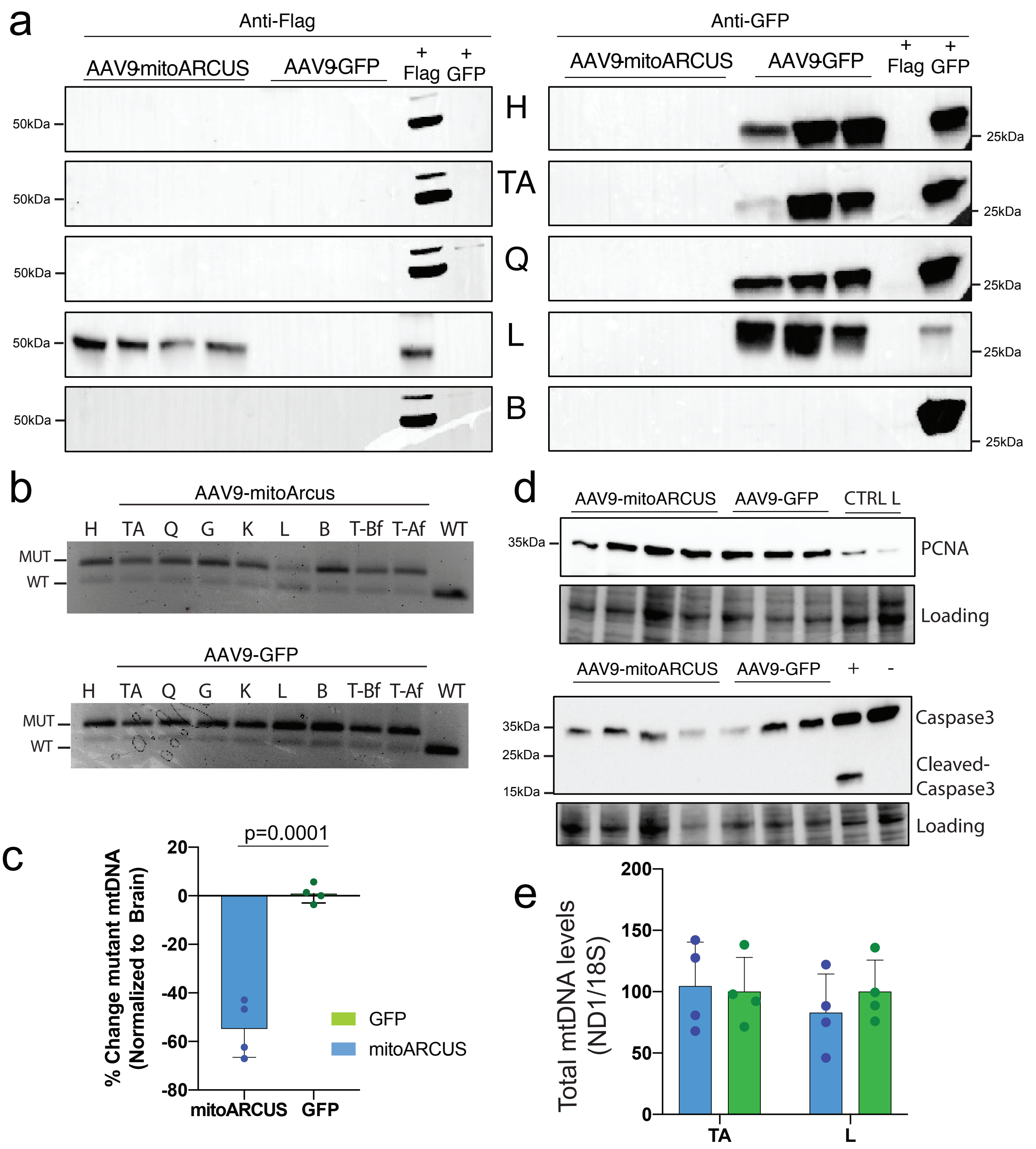

Supplementary Fig S3: AAV9-mitoARCUS effect in young treated mice 10 days Post Injection a) Western blots of homogenates probed with Flag antibody for AAV9-mitoARCUS and GFP for AAV9-GFP samples for heart (H), tibialis anterior (TA), quadriceps (Q), liver (L) and brain (B). This experiment was repeated twice. b) Representative RFLP analysis of DNA samples from an animal injected with AAV9-mitoARCUS and an animal injected with AAV9-GFP 10 days PI in heart (H), tibialis anterior (TA), quadriceps (Q), gastrocnemius (G), kidney (K), liver (L), brain (B), tail before injection (T-Bf), and tail after injection (T-Af). This analyses was performed for each animal. c) Quantification of heteroplasmy shift shown as percent change in heteroplasmy in liver, normalized to brain tissue, from results obtained with RFLP “last cycle hot” PCR analysis. d) Western blots of liver homogenates of animals injected with AAV9-mitoARCUS (n=4) and AAV9-GFP (n=3). Expression of PCNA shown in blot with control livers from healthy adult mice. Expression of Caspase3 and Cleaved-Caspase3 shown in blot with positive control representing MEFs treated with staurosporine, negative control representing MEFs not treated with staurosporine. Blots are shown along with total protein loading. This experiment was repeated twice. e) Quantification by qPCR of total mtDNA levels were measured in liver (L) and tibialis anterior (TA) at 10 days PI using ND1 mitochondrial primer/probes normalized to 18S (nuclear DNA). Data are mean ± SEM of n=4. Statistical analysis was performed using two-tailed student’s t-test.

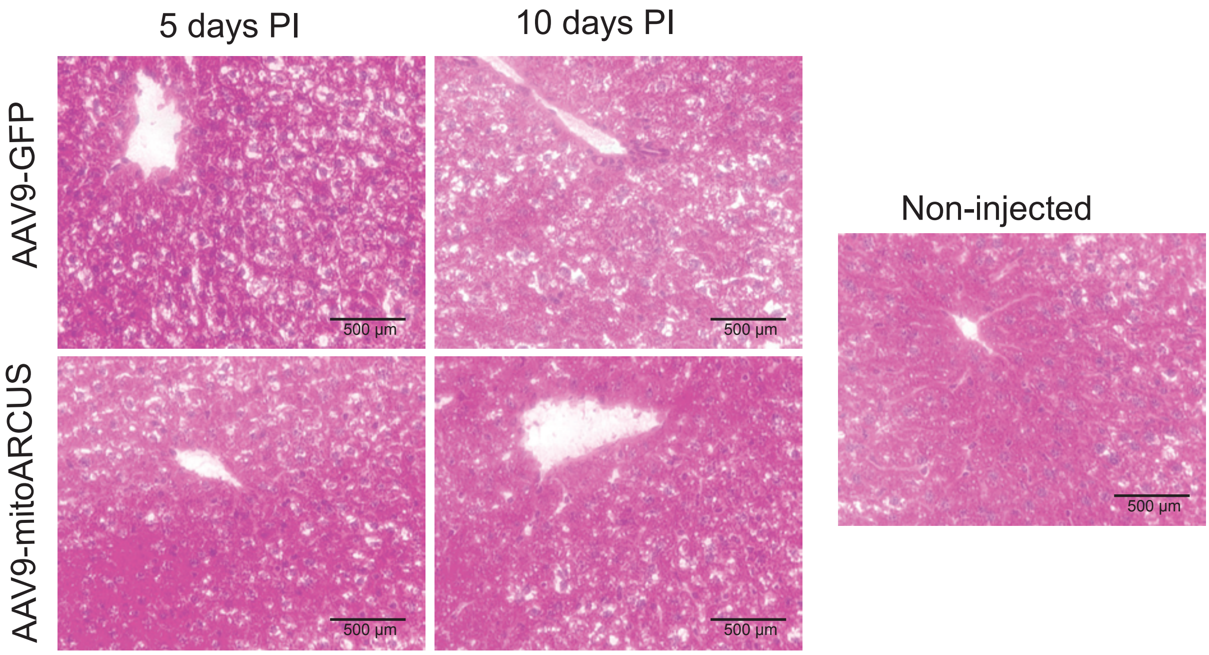

Supplementary Fig S4: Liver histology at 5 and 10 days PI Liver samples were embedded in OCT and snap-frozen. Samples were sliced to 7µm thickness and stained for H&E. Bar= 500µm. This experiment was repeated twice with identical results.
