# Supplementary material for: Mitochondrial targeted meganuclease as a platform to eliminate mutant mtDNA in vivo
Source: Nat Commun. 2021 May 28;12:3210. doi: 10.1038/s41467-021-23561-7 (PMC8163834; doi:10.1038/s41467-021-23561-7)
Supplement: Supplementary file 3 — Reporting Summary [file 41467_2021_23561_MOESM3_ESM.pdf]

nature research

Corresponding author(s): Carlos T. Moraes

Last updated by author(s): Apr 8, 2021

Reporting Summary

Nature Research wishes to improve the reproducibility of the work that we publish. This form provides structure for consistency and transparency in reporting. For further information on Nature Research policies, see our Editorial Policies and the Editorial Policy Checklist.

Statistics

For all statistical analyses, confirm that the following items are present in the figure legend, table legend, main text, or Methods section.

n/a	Confirmed	
☐	☒	The exact sample size (n) for each experimental group/condition, given as a discrete number and unit of measurement
☐	☒	A statement on whether measurements were taken from distinct samples or whether the same sample was measured repeatedly
☐	☒	The statistical test(s) used AND whether they are one- or two-sided<br>*Only common tests should be described solely by name; describe more complex techniques in the Methods section.*
☒	☐	A description of all covariates tested
☒	☐	A description of any assumptions or corrections, such as tests of normality and adjustment for multiple comparisons
☐	☒	A full description of the statistical parameters including central tendency (e.g. means) or other basic estimates (e.g. regression coefficient) AND variation (e.g. standard deviation) or associated estimates of uncertainty (e.g. confidence intervals)
☐	☒	For null hypothesis testing, the test statistic (e.g. F, t, r) with confidence intervals, effect sizes, degrees of freedom and P value noted<br>*Give P values as exact values whenever suitable.*
☒	☐	For Bayesian analysis, information on the choice of priors and Markov chain Monte Carlo settings
☒	☐	For hierarchical and complex designs, identification of the appropriate level for tests and full reporting of outcomes
☒	☐	Estimates of effect sizes (e.g. Cohen's d, Pearson's r), indicating how they were calculated

Our web collection on statistics for biologists contains articles on many of the points above.

Software and code

Policy information about availability of computer code

Data collection	BioRad Chemidoc (western and northern blots), Cylcone phosphor-imaging system (Perkin Elmer, RFLP), Seahorse XFp Extracellular Flux Analyzer (Seahorse Bioscience)
Data analysis	BioRad Image Lab (version 6.0.1), OptiQuant software Version 5.0 (Perkin Elmer), GraphPad Prism 7 and 8

For manuscripts utilizing custom algorithms or software that are central to the research but not yet described in published literature, software must be made available to editors and reviewers. We strongly encourage code deposition in a community repository (e.g. GitHub). See the Nature Research guidelines for submitting code & software for further information.

Data

Policy information about availability of data

All manuscripts must include a data availability statement. This statement should provide the following information, where applicable:

- Accession codes, unique identifiers, or web links for publicly available datasets
- A list of figures that have associated raw data
- A description of any restrictions on data availability

All data is provided

Field-specific reporting

Life sciences study design

All studies must disclose on these points even when the disclosure is negative.

Sample size	No sample size calculation was performed. n=4 for each condition was used because of restrictions of viral titer received.
Data exclusions	N/A
Replication	Measurements were done in triplicate to ensure result reliability.
Randomization	Sibling mice were injected randomly with treatment or control.
Blinding	No blinding was necessary, as mice were injected with treatment (AAV9-mitoARCUS) or control (AAV9-GFP).

Reporting for specific materials, systems and methods

We require information from authors about some types of materials, experimental systems and methods used in many studies. Here, indicate whether each material, system or method listed is relevant to your study. If you are not sure if a list item applies to your research, read the appropriate section before selecting a response.

Materials & experimental systems

n/a	Involved in the study	
☐	☒	Antibodies
☐	☒	Eukaryotic cell lines
☒	☐	Palaeontology and archaeology
☐	☒	Animals and other organisms
☒	☐	Human research participants
☒	☐	Clinical data
☒	☐	Dual use research of concern

Methods

n/a	Involved in the study	
☒	☐	ChIP-seq
☐	☒	Flow cytometry
☒	☐	MRI-based neuroimaging

Antibodies

Antibodies used	Antibodies used were mouse monoclonal Flag (F3165, Sigma) (1:1000), mouse monoclonal GFP (75-131, UC Davis) (1:1000), mouse monoclonal MTCO1 (ab14705, abcam) (1:1000), mouse monoclonal NDUFB8 (ab110242, abcam) (1:750), mouse monoclonal Tubulin (T9026, Sigma) (1:20,000), rabbit polyclonal Caspase-3 (#9662, Cell Signaling) (1:1000), and mouse monoclonal PCNA (PC10 #2586, Cell Signaling) (1:2000).
Validation	*Describe the validation of each primary antibody for the species and application, noting any validation statements on the manufacturer's website, relevant citations, antibody profiles in online databases, or data provided in the manuscript.*

Eukaryotic cell lines

Policy information about cell lines

Cell line source(s)	Mouse embryonic fibroblasts (MEFs) derived from C57BL/6J heteroplasmic mice carrying m.5024C>T point mutation for analysis of mtDNA depletion, heteroplasmy change, and oxygen consumption rates , HEK293T (ATCC CRL-3216) for protein expression, HeLa (CCL-2 Cells, ATCC) for immunofluorescence
Authentication	MEFs had the expected mtDNA genotype. HEK293T and HeLa Cells were unauthenticated
Mycoplasma contamination	Cells were regularly checked for and tested negative for mycoplasma.
Commonly misidentified lines (See ICLAC register)	*Name any commonly misidentified cell lines used in the study and provide a rationale for their use.*

Animals and other organisms

Policy information about studies involving animals; ARRIVE guidelines recommended for reporting animal research

Laboratory animals	C57BL/6J heteroplasmic mice carrying m.5024C>T point mutation
Wild animals	N/A
Field-collected samples	N/A

Ethics oversight | All animal procedures were approved by the University of Miami Animal Care and Use Committee and by IACUC.

Note that full information on the approval of the study protocol must also be provided in the manuscript.

Flow Cytometry

Plots

Confirm that:

- [x] The axis labels state the marker and fluorochrome used (e.g. CD4-FITC).
- [x] The axis scales are clearly visible. Include numbers along axes only for bottom left plot of group (a 'group' is an analysis of identical markers).
- [ ] All plots are contour plots with outliers or pseudocolor plots.
- [ ] A numerical value for number of cells or percentage (with statistics) is provided.

Methodology

Sample preparation	Heteroplasmic MEFs were transfected using GenJet DNA In Vitro Transfection Reagent (Ver. II) (SL100489, SignaGen Laboratories) using manufacturer's protocols. We transfected cells plated in a T75 flask at 80% confluence with 30 µg plasmid total, in a 2:1 ratio of mitoARCUS CF or CSF plasmid (20ug) to GFP plasmid (10ug).
Instrument	FACS Aria IIU, gating on single cell fluorescence using a 488nm laser and 505LP, 530/30 filter set for GFP expression
Software	BD FACSDiva software, Version V8.0.2
Cell population abundance	"Green" cells made up 11-20% of the transfected cell population (MEFs). The remainder of the cells were sorted into the "Black" cell population.
Gating strategy	Transfected MEF cells were sorted into Black populations (no GFP expression) and Green populations (GFP expression). Untransfected control cells were also passed through the cell sorter in order to expose cells to same conditions.

- [x] Tick this box to confirm that a figure exemplifying the gating strategy is provided in the Supplementary Information.
